# Psychological Well-Being and Intra-personal Conflicts in Adolescence

**DOI:** 10.11621/pir.2021.0309

**Published:** 2021-09-30

**Authors:** Larisa A. Golovey, Marina V. Danilova, Irina A. Gruzdeva, Liudmila V. Rykman

**Affiliations:** Saint-Petersburg State University, Saint-Petersburg, Russia

**Keywords:** Psycho-Emotional Well-Being (PEW), Intra-Personal Conflicts (IPC), Motivation-Value Conflict (MVC), Self-Esteem Conflict (SEC)

## Abstract

**Background:**

Adolescence is a period characterized as transitional and as such, it is full of complications and conflicts. Research of Intra-Personal Conflicts in connection with Psycho-Emotional Well-being (PEW) comprising three kinds of indicators: personality, cognitive-evaluative and emotional represents new scientific approach. This approach provides the opportunity to define the role of PEW in Intra-Personal Conflicts: Motivation Value Conflict (MVC) and Self-Estimate Conflict (SEC).

**Objective:**

Our aim was to study the severity of MVC and SEC, the interrelationship of these types of conflicts, and their connection with various PEW components.

**Design:**

237 high school students (ages 15–18; 99 boys, 138 girls) were surveyed. Tests of MVC, the Self-Estimate Scale (SE), and the Level of Aspiration Scale (LA) were applied to measure the conflicts. The Scale of Psychological Well-Being, the Scale of Life Satisfaction, and the Dominant Emotional States Test were employed to measure PEW.

**Results:**

The study revealed a high prevalence of Intra-Personal Conflicts in the sample. The adolescents all had high levels of Intra-Personal Conflicts; changes were found in all three blocks of PEW. In the group with a high level of MVC, the levels of Environmental Mastery and Self-Acceptance were significantly lower. Having high level of SEC went along with decreases in most indicators of the personal and cognitive-evaluative components of PEW: decreasing of Cheerfulness, Active Attitude to Life Situation and Life Satisfaction; there were changes in emotional blockage, including decreases in Stability and Emotional Tone, and increases in Despondency, Tension, and Anxiety.

**Conclusion:**

The study found the prevalence of Intra-Personal Conflicts in the adolescents. We showed that the personality and cognitive-evaluative components of PEW played the role of conflict moderators, while the emotional components were manifested as intra-personal conflict.

## Introduction

Over the last decade, the problem of Psychological Well-Being (PEW) has attracted more and more attention, not only from psychologists, sociologists, and educators, but also from politicians, economists, governmental authorities, and the whole international community. The interdisciplinary nature of this problem has been indicated by D.A. Leontiev (2020). It should be noted that psychology does not have a generally accepted scientific concept of “Psychological Well-Being”; moreover, some approaches to its formulation and structural determination are subject to debate.

Two approaches can be considered the most traditional: the hedonistic and the eudaimonic. N. Bradburn and E. Diener are the founders of the hedonistic approach to research on subjective Well-Being (Diener, Oishi, & Tay, 2018). The proponents of this approach consider the experience of Happiness, Life Satisfaction, Positive Thinking, and Social Behavior to be the most common indicators of Well-Being (Diener et al., 2018; Shamionov, 2004). In the eudaimonic approach, Psychological Well-Being is defined as a basic subjective construct that reflects the individual’s perception and assessment that they are functioning at their highest potential, and are seeking Self-Actualization. This construct includes Autonomy, Environmental Mastery, Focus on Personal Growth, Positive Relationships, Life Goals, and Self-Acceptance (Ryff & Singer, 2008). Modern psychologists tend to merge both approaches and view them as aspects of an overall picture of Well-Being (Henderson & Knight, 2012). The term Psycho-Emotional Well-Being is also used, which usually means resistance to stress, and lack of Depressiveness or excessive Anxiety (Podolskiy, Karabanova, Iodobaeva, & Heymans, 2011; Reeve, 2014).

We based our study on the concept of Psycho-Emotional Well-Being (PEW), which combines these approaches, using such notions as personality (Psychological Well-Being,) (Ryff et al., 2008), cognitive-evaluative state (Life Satisfaction), and emotional-affective condition (sustainable emotional states) (Troshikhina & Manukyan, 2017). This approach provides the most complete definition of individual inner Well-Being. From this standpoint, researchers study the influence of such factors as age, gender, self-control, family crises, social support, contact with nature, etc. on Psychological Well-Being (Ronen, Hamama, Rosenbaum, & Misheli-Yarlap, 2016; Golovey (Ed.), 2020).

The relationship between Psychological Well-Being and Intra-Personal Conflicts and their role in personality development is also subject to debate. Some scientists (Vygotsky, 1983; Bozhovich, 1968; Asmolov, 1990) consider conflicts as a factor which stimulates Personality Development, while others stress their ambiguous, and often negative, impact on the individual’s emotional state and development (Borozdina & Zaluchenova, 1993; Vasilyuk, 1984; Fantalova, 2001, 2015).

Intra-Personal Conflict is viewed as a clash of opposing trends, interests, ideas, and aspirations within an individual. Researchers pay the most attention to Motivation-Value (MVC) and Self-Estimate conflicts (SEC). Motivation-Value Conflicts (MVC) are operationalized through the study of the ratio of values an individual considers vital and their availability (Fantalova, 2001, 2015). Much research has been devoted to the structure of values, and their hierarchy. Researchers have uncovered features of gender value conflicts, their relationship with Mature Inner World, Creativity, Protective Inactivity, and Difficulty in Life Agenda Implementation (Zhuravlev & Drobysheva, 2010; Vartanova, 2014; Fedotova, 2017; Golovey & Gruzdeia, 2017). Relationships between an individual’s Value Orientation, Subjective Well-Being, and Emotional Orientation have also been highlighted (Shamionov, 2004).

Self-Estimate Conflict (SEC) is identified as a conflict between an adolescent’s level of Self-Esteem (SE) and Level of Aspiration (LA). Self-Esteem as a person’s general evaluation of his or her value is expressed as either positive or negative Self-Orientation. Scientists point to the special role of Self-Esteem in adolescence, and its connection with Academic Performance and Mental Health (Leavitt, Covarrubias, Perez, & Fryberg, 2015; Tagai, 2017; Minev et al., 2018). It has been shown that inadequate Self-Esteem can act as a predictor of Aggressiveness, Anxiety, Frustration, and decreased Life Satisfaction. (Diener, E. & Diener, M., 1995; Paradise & Michael, 2005; Kuzmina, E. & Kuzmina, Z., 2018; Vodyakha, S. & Vodyakha, Y., 2019).

However, there is far less information on the links between MVC, Personality Development, and Well-Being. Moreover, there are indications of these conflicts’ negative impact on adolescents’ Socialization and Adaptation. (Prikhozhan & Tolstikh, 2016). Some studies suggest that a discrepancy between levels of Self-Esteem and Level of Aspiration in early adolescence can lead to an increase in Anxiety and result in Somatic disorder. (Borozdina & Zaluchenova, 1993; Sidorov, 2007; Pukinska, 2008).

As our analysis showed, the relationship of MVC and SEC with Psychological Well-Being indicators (personality, cognitive-evaluative, and emotional) still remains understudied.

## Methods

### Measurements

The following tests were administered: the Intra-Personal Conflicts study (AVR-Availability Value Ratio) (Fantalova, 2001); the Dembo-Rubinstein Self-Assessment and Level of Aspiration Scale (modified by Prikhozhan, 1988); and the Psychological Well-Being Scale by C. Ryff (adapted by Zhukovskaya & Troshikhina, 2011). The instrument consists of six scales: Autonomy; Environmental Mastery; Personal Growth; Positive Relations with Others; Purpose in Life; and Self-Acceptance. We also used E. Diener’s Life Satisfaction Scale (adapted by Osin & Leontiev, 2008), and the Testing Dominant Emotion States (by Kulikov, 1998), which consists of the following scales: Active vs. Passive Attitude toward one’s Life Situation; Cheerfulness vs. Despondency; Emotional Tone (high or low); Relaxation vs. Tension; Tranquility vs. Anxiety; Stability vs. Instability of Emotional Tone; and Satisfaction vs. Dissatisfaction with Life. All the methods used in the study were adapted and tested on adolescent samples and had normalized scores.

### Participants

The study sample was comprised of 237 high school and gymnasium students, ages 15 to 18 (99 boys, 138 girls). The study was conducted in regular class sessions in 2018. The parents were given an explanation of the purpose of the study, and informed consent was obtained from them.

### Procedure

#### Study Objectives

To reveal the severity of MVC and SEC as varieties of Intra-Personal Conflicts in adolescence and find out the relationship of these conflict types with each other and various PEW substructures.

#### Hypotheses

We proceeded from the following assumptions: 1) Various types of Intra-Personal Conflicts, in particular MVC and SEC, can be interrelated; 2) Pronounced Intra-Personal Conflicts may have two-way relationships with some levels of PEW; and 3) Different substructures of PEW can be interconnected with Intrapersonal Conflicts in different ways. We assumed that personal and cognitive-evaluative substructures can act as predictors of conflicts, either weakening or strengthening them. The emotional substructures of PEW act as consequences of conflicts.

## Results

The data analysis showed that the sample’s general level of Psychological Well-Being (M = 188.83; σ = 20.01) and Life Satisfaction (M = 23.46; σ = 6.66) corresponded to mean values, and in the given sample, reflected a generally favorable picture of Maturation. However, there was a high variability of indicators where the mean values did not reflect its individual characteristics.

The comparative analysis of the frequency of conflict manifestations revealed that both types of conflict were prevalent, with high levels of SEC accounting for the greater number of respondents (52%), and that of MVC for 19.3%.

The mean value of the total MVC index in the sample was 34.9 points, σ = 15.24, which indicated an insignificant degree of Dissociation and a gap between the Values and their Availability. Draw your attention to the wide variability of the MVC index (value scores ranged from — 5 to 69), which significantly exceeded the standard values obtained by the author of the method (Fantalova, 2015). All its indicators were expressed in the structure of MVC (*Table 1*).

**Table 1:** Indicators of value imbalance (score in points), n 237

Indicators of concordance in value	M	σ	Skewness	Std.Error	Kurtosis	Std.Error	Cronbach’s alpha
Inner Conflict (IC)	12.63	8.69	.342	.158	-.584	.315	.645
Internal Vacuum (IV)	10.67	9.05	.407	.158	-1.041	.315	.738
Neutral Zone (NZ)	11.80	4.39	.125	.158	.358	.315	.632

*Note. M = Mean; σ = Standard Deviation.*

The sample mean data indicated that Neutral Zones (65.5%) were expressed in the Internal Conflict framework; these zones are characterized by Values Coincidence and their Availability for Satisfaction. Internal Vacuums which means Redundancy of Availability in the Absence of its value prevailed in 15.19% of respondents. More than 19% percent (19.3%) of respondents had a pronounced Internal Conflict due to the Unavailability of Desired Values. Valid differences in Value/Availability ratio were revealed in 8 out of 12 life spheres (with significance level p ranging from .004 to .000).

The most significant discrepancies between Values and their Availability were identified by such indices as Happy Family Life, Love, and Financially Secure Life, whereas a decrease in incentive motivation was noted in Beauty of Nature and Art, Active life, Creativity, and Cognition, which are characteristic of Internal Vacuum. The values of Health, Friends, Freedom, Challenging Work, and Self-Confidence were confined to the Neutral Zone.

Given the high variability of the indicators, a further analysis was carried out on groups with different levels of MVC severity. The group division was based on a Conflict Integral indicator, which is the sum of differences between a Value and its Availability in various spheres of life. An index equal to or exceeding 50 points meant the unavailability of significant values and indicated an individual’s Motivation Disintegration, Deep Dissatisfaction, and Blockage of Basic Necessities. The two groups we distinguished were: Group 1, which consisted of adolescents with a low MVC level (144 respondents), and Group 2, which had medium or high MVC levels (94 respondents). Girls outnumbered boys in Group 2 (p = .005). These groups differed in their total MVC indices (p = .000) and in conflict structure (*Table 2*).

**Table 2:** Differences in a conflict structure and indicators of psycho-emotional Well-Being in groups with different levels of MVC

Conflict indicators and PEW Valid	Group 1		Group 2		Mann-Whitney U-test	(p) Significance
	M	σ	M	σ		
SEC, Confidence	27.55	23.03	33.14	22.9	5668.5	.04
Total Index MVC	24.5	7.08	50.7	8.3	33.00	.000
Inner Conflicts, %	10.52	8.15	27.41	8.76	969.00	.000
Internal Vacuums, %	6.80	7.0	27.9	8.4	503.00	.000
Neutral Zones, %	82.69	12.6	44.6	12.1	195.50	.000
Environmental Mastery	29.2	4.5	27.4	5.06	5482.50	.016
Personal Growth	33.2	5.0	34.4	4.2	5650.50	.038
Self-Acceptance	31.4	6.2	28.7	6.9	5266.50	.005

*Note. M = Mean. σ = Standard Deviation.*

As you see in the table, the MVC for the adolescents in Group 1 was distinguished by 2.7 times fewer Internal Conflicts (p = .000), more Neutral Zones (p = .000), and fewer Internal Vacuums (p = .000), while in Group 2, the percentage of Internal Conflicts (p = .000) and Internal Vacuums (p = .000) was significantly higher, with fewer Neutral Zones (p = .000). These indicators suggest the Disintegration of Motivation, as a high level of conflict zones coexisted with poorly represented zones with Value-Availability concordance. There were more zones with a Low Level of Values and High Availability of them. This suggests that these adolescents have what they do not need and miss what is really meaningful to them.

A comparison of the Values/Availability ratio in different spheres of life revealed a lower level of divergence of values Active Life (p = .000), Cognition (p = .000), Beauty of Nature and Art (p = .000), and Creativity (p = .000) and their availability in Group 2, along with reduced motivation (Mann-Whitney U-test). At the same time, Friends (p = .000), and Family (p = .000) were the most conflicted spheres. Thus, in the adolescents with pronounced MVC, there was a change in conflict structure and range of represented life spheres. It is also noteworthy that they had SEC in the area of Self-Confidence.

The analysis of differences in PEW indicators between the groups revealed differences in personality indicators: in Group 2, the Level of Environmental Mastery (p = .016) and Self-Acceptance (p = .005) was lower, while the Level of Personal Growth pursuit was higher. The differences in parameters of dominant emotional states were negligible.

The analysis of Self-Esteem and Level of Aspiration levels revealed that the sample fit within a framework of the average statistical norm (Self-Esteem, M = 61.8; Level of Aspiration, M = 85.05), with high individual variability (from 0 to 100), and lower values among girls. Partial indicators of Self-Esteem fluctuated from 59.6 points to 72.6 points on different scales and indicated its mean level. The adolescents rated Intelligence and Character the highest and gave lower scores to their Peers’ Authority, Self-Esteem, and Manual Skills. Scores on the Level of Aspiration demonstrated high values (from 75 to 89 points on all scales), which reflected the optimism of both the boys and girls about their capabilities. Like for Self-Esteem, the results showed very large individual variation (min — 15 points, max — 100 points). The highest Level of Aspiration was in Intelligence and Appearance; all other areas were evenly distributed.

Under our methodology, an Indicator of Conflict was considered to be a discrepancy between the adolescent’s Level of Aspiration (LA) and Self-Esteem (SE) (less than 8 points or more than 22 points) (Prikhozhan, 1988). On this basis the sample was subdivided into three groups: the first was made up of respondents with no SEC expressed (113 respondents). The second group consisted of respondents with a pronounced SEC (the difference between AL and SE is more than 22points — 114 respondents). These two groups were balanced by gender. The third group consisted of young men only, whose SEC indicator was less than 8 points (10 respondents). The representatives of the third group demonstrated a rare conflict between decreased Level of Aspiration and increased Self-Esteem, which reflects a state of Protective Inactivity. To identify differences between the groups, we applied a one-way analysis of variance. In further analysis, the results of the third group were not analyzed due to its small size and gender homogeneity. The differences between groups 1 and 2 in SE and AL and all scales of the methodology (p = .000) are presented in *Table 3.*

**Table 3:** Differences in indicators of psycho-emotional Well-Being between groups with different levels of SEC (results of one-way analysis of variance)

SEC and PEW Indices	Group I (n-113)		Group II (n-114)		Mean M, (i–j)	(p) Significance
	M	σ	M	σ		
SEC (Total Index)	15.40	3.7	33.5	9.9	18.1	.000
Life Satisfaction	23.9	6.6	21.2	6.5	–2.68	.008
Autonomy	32.4	5.6	29.8	5.7	–2.56	.003
Environmental Mastery	29.8	4.7	27.1	4.6	–2.64	.000
Positive Relations with Others	33.1	5.9	30.3	5.6	–2.77	.002
Self-Acceptance	31.9	6.1	28.8	6.3	–3.03	.002
PEW (total Index)	194.2	20.08	179.2	19.2	–15.06	.000
Activeness-Passiveness	47.2	10.9	44.4	10.2	3.49	.004
Cheerfulness-Despondency	50.5	9.2	44.9	9.2	5.55	.000
Relaxedness - Tension	50.5	10.8	45.9	10.2	–4.63	.005
Satisfaction– Dissatisfaction	53.1	9.9	49.2	11.2	–3.94	.002

*Note. M = Mean; σ = Standard Deviation.*

The analysis of the differences in PEW indices between the groups showed that they differed in Life Satisfaction (p = .008), Autonomy and Environmental Mastery (p = .000), and Positive Attitudes and Self-Acceptance (p = .002), as well as in the Total Indicator of Psychological Well-Being (p = .000). All these indicators were significantly lower in adolescents with a high SEC level. Some differences were found in dominant emotional states. Group 2 adolescents showed lower Activity (p = .004), lower Cheerfulness (p = .000), decreased Life Satisfaction (p = .005), and increased Tension (p = .002). Thus, the conflict of Self-Esteem affected all three constituents of PEW: personality, cognitive-evaluative, and emotional.

In order to confirm the results obtained in the analysis of differences, we undertook correlation and regression analyses. Correlation analysis was carried out on the groups with a high level of conflict. Among MVC indicators, the Level of Conflict in Freedom was found to be the most interconnected with negative links of PEW indices with Life Satisfaction, Positive Relationships, Self-Acceptance, Total Level of Well-Being, Emotional Stability, and Satisfaction as a sustainable state (six connections at p .001 – .005). The Conflict in Creativity had four negative connections: Environmental Mastery, Self-Acceptance, Total Well-Being, and Tranquility (p = .001). Dissatisfaction with Family was directly related to Environmental Mastery and Total Level of Psychological Well-Being (p = .005). The total indicator of Internal Conflict formed two negative connections, one with Autonomy and one with positive Self-Image. The high value of the parameter Neutral Zones was directly related to positive Self-Image (p = .001); the parameter Internal Vacuums was interconnected with passive Life Attitude (p = .005). Therefore, various PEW indices were found to be interrelated with manifestations of MVC. Conflicts in Freedom and Creativity tended to be most integrated into the PEW framework.

The analysis of SEC relationships revealed 21 negative connections with all PEW indices. Various indicators of SEC tended to be linked with Life Satisfaction, Self-Acceptance (eight connections at p .017 – .001), Environmental Mastery, Autonomy, Total Level of Psychological Well-Being (nine connections at p .022 – .000), and Life Goals (one connection) (p = .002). Relationships with Emotional states (three connections at p .028 – .000) indicated that an increase in SEC was accompanied by a decrease in Vigor, Emotional Stability, and Self-Acceptance. Indicators of Total SEC Index and Self-Confidence Conflict were most involved in the structure of connections with PEW.

Regression analysis was also carried out in the group with a high level of conflicts. It is significant that in the group with SEC, in addition to the size of the discrepancy between the Level of Aspiration and Self-Esteem, indicators of MVC were found to be dependent variables, and in the group with high indicators of MVC, SEC variables were found.

We created four models that re" ected the relationship of PEW with various types of conflicts: two models showed the relationship of PEW with SEC, and two models, the relationship of MVC with PEW (*Table 4*). SEC was described by the model as accounting for 31.9% of the variance. Total Level of Psychological Well-Being (β = –.317; p = .025) and Environmental Mastery (β = –.281; p = .045) were the predictors in this model. The Total SEC indicator was included as a dependent variable in the second model (with variance 13.9%), with the independent variables being Life Satisfaction (β = –.296; p = .001) and Autonomy (β = –.317; p = .025). In the third model, Self-Acceptance (β = .217; p = .027), Environmental Mastery (β = .293; p = .005), and Positive Relations with Others (β = –.234; p = .011) predicted 19% of the variance on Neutral Zones. The independent variables of Self-Acceptance (β = –.191; p = .05), Positive Relations with Others (β = .250; p = .008), and Environmental Mastery (β = –.261; p = .013) predicted 16% of the variance for the Total Value Conflict index in the fourth model.

**Table 4:** The impact of PEW on Conflicts indicators

Dependent Variable	R Square	Predictors	β	p
Model 1				
SEC	.319	Total Level of Psychological Well-Being	–.317	.025
		Environmental Mastery	–.281	.045
Model 2				
SEC	.139	Life Satisfaction	–.296	.001
		Autonomy	–.317	.025
Model 3				
Neutral Zones	.190	Self-Acceptance	.217	.027
		Environmental Mastery	.293	.005
		Positive Relations with Others	–.234	.011
Model 4				
MVC (Total Index)	.160	Self-Acceptance	–.191	.05
		Positive Relations with Others	.250	.008
		Environmental Mastery	–.261	.013

At the next stage of the regression analysis, the dominant emotional states were included as dependent variables, while the indicators of Conflicts were the independent factors. Four models were obtained (*Table 5*). In the first model (variance 37%) Internal Conflicts of Family Values (β = –.342; p = .005), Cognition (β = –.328; p = .000), Freedom (β = –.253; p = .005), Total MVC index (β = –.172; p = .048) and SEC in Skills (β = .224; p = .000) predicted the dependent variable Cheerfulness vs. Despondency. In the second model, the dependent variable Relaxation vs. Tension (variance 23.6%) was predicted by Internal Conflicts in Health (β = –.342; p = .054), Freedom (β = –.384; p = .021), and Family (β = –.441; p = .012). In the third model, the dependent variable Tranquility vs. Anxiety (variance 21.1%) was predicted by Internal Conflict in Beauty of Nature and Art (β = .343; p = .000), Creativity (β = .195; p = .05), and SEC in Self-Confidence (β = –.235; p = .018). In the fourth model, the dependent variable Satisfaction vs. Dissatisfaction with Life (variance 10.3%) was predicted by the levels of Internal Conflicts in Freedom (β = .292; p = .002), and Family (β = –.216; p = .021).

**Table 5:** The impact of Conflicts indicators on Dominant Emotional States

Dependent Variable	R Square	Predictors	β	p
Model 1				
Cheerfulness- Despondency	.370	Internal Conflict of Family Values	–.342	.000
		Internal Conflict of Cognition Values	–.382	.000
		Internal Conflict of Freedom Values	–.253	.005
		MVC (Total index)	–.172	.048
		SEC in Skills	.224	.005
Model 2				
Relaxedness- Tension	.236	Internal Conflict in Health Values	–.342	.054
		Internal Conflict in Freedom Values	–.384	.021
		Internal Conflict in Family Values	–.441	.012
Model 3				
Tranquility- Anxiety	.211	Internal Conflict in Beauty of Nature and Art Values	.343	.000
		Internal Conflict in Creativity Values	.195	.050
		SEC in Self-Confidence	–.235	.018
Model 4				
Satisfaction- Dissatisfaction with Life	.103	Internal Conflicts in Freedom Values	–.292	.002
		Internal Conflict in Family Values	–.216	.021

The results of regression analysis showed that SEC and MVC were a part of all models mentioned above. Correlation analysis revealed 13 connections between these conflicts. The conflicts most related to total SEC in Financial Satisfaction were SEC in Intelligence, Authority, Confidence, Appearance, and Character indices (six connections, p = .001); SEC in Intelligence and Skills had four negative connections with MVC through such indices as Friends, Family, Love, and Health (p = .005; .001).

## Discussion

The results of our PEW analysis revealed an overall positive picture of the respondents’ maturation over high individual variability, which was consistent with the data obtained from other samples (Pavlova & Benkova, 2016; Golovey & Danilova, 2019). Our study of the most common conflicts in adolescence revealed a widespread prevalence of MVC and SEC. The variability of the MVC data, which significantly exceeded the results of a 2001 normative sample (Fantalova, 2001), might indicate an increase in Financial Family Stratification, which was reflected in an increase in MVC, which indicates the initial stages of Value Disintegration. The importance of Financial Satisfaction was also confirmed by the presence of a large number of correlations between this indicator and manifestations of both Total MVC level and SEC level.

The respondents with high levels of MVC were characterized by a change in MVC structure. In the framework of conflict, an increase in conflict zones was combined with a decrease in Neutral Zones and an increase in internal vacuum zones. This indicated Unavailability of significant values, an increase in Value /Availability concordance, and a decrease in a number of zones of Motivation Level of Development such as Creativity, Beauty of Nature and Art, Cognition, and Active Life. The respondents with a high level of SEC showed a decrease in the Total and in all indicators of this conflict.

The analysis of differences in PEW indicators revealed that in the groups with a high level of MVC, Environmental Mastery and Self-Acceptance were significantly lower, but Desire for Personal Growth was higher, which may indicate a motivating role of MVC and is consistent with the results (Golovey & Gruzdeva, 2020) for adolescents. The group with a high level of SEC was characterized by a decrease in all indicators of Personality and Cognitive-Evaluative PEW indices, coupled with a decrease in Vigor, Activity, Life Satisfaction, and an increase in Tension, *i.e.,* all MVC indices are involved in a SEC conflict.

Correlation analysis confirmed that an increase in the severity of Internal Conflicts was correlated with an increase in the indicators of Personality and Cognitive-Evaluative PEW indices, and was also accompanied by the changes in the emotional part of PEW. These changes in the emotional component were manifested by a decrease in Emotional Stability, Activity, and an increase in Depression, Tension, and Anxiety, which correlates with the “Anxiety Triad” described in SEC (Borozdina & Zaluchenova, 1993; Sidorov, 2007; Pukinska, 2008). In our study, similar changes were observed in both conflicts.

We used two strategies to conduct the regression analysis, In the first strategy, the Personality indicators and Cognitive-Evaluative components of PEW were taken as independent variables, whereas indicators of conflicts were taken as dependent variables. The results of this strategy application showed that MVC and SEC predictors were low indicators of Life Satisfaction, Autonomy, Environmental Mastery, and a decrease in Overall Assessment of Psychological Well-Being. In MVC conflict, Self-Acceptance, Environmental Mastery, Positive Attitudes were the factors that contributed to an increase in a number of Neutral Zones, thus reducing Conflict Tensions. In the second strategy, the independent variables were indicators of Conflicts, while the dependent ones were dominant Emotional States. It can be seen from the regression models that Family Dissatisfaction, Cognition, Beauty of Nature and Art, Freedom, and Health in MVC and SEC were linked to an increase in Confidence, while a decrease in Motivation worked as a predictor of Despondency, Tension, Anxiety, and Dissatisfaction.

Therefore, the results showed the role of different PEW indices in the Intra-Personal Conflicts of adolescence. Personality and cognitive-evaluative components played the part of predictors and possible moderators of conflicts. This view is consistent with the understanding of PEW personality parameters as sustainable personality traits which reThect its psychological maturity as a result of ontogenesis, and act as moderators in difficult life situations (Zhuravlev & Sergienko (Eds.), 2007; Golovey (Ed.), 2014; Manukyan & Troshikhina, 2016; Golovey et al., 2019). Cognitive-evaluative indicators, especially Life Satisfaction, are considered as a sustainable property with a high contribution of the genetic factor (Diener et al., 2018). Emotional components, reflecting everyday dominant emotional states, are manifestations of the effects of intra-personal crises and conflicts. Earlier, such results were obtained in a study of SEC, which revealed the “Risk Triad,” that is, the Discrepancy between high levels of SE and LA, Increased Anxiety, and Somatic Diseases (Sidorov, 2007; Pukinska, 2008).

From our study we concluded that negative changes affected not only Anxiety, but also other emotional states; they were observed in both SEC and MVC. The internal relationship between the two above-mentioned Intra-Personal Conflicts was most clearly manifested in the connection between the severity of SEC in Intelligence, Authority, Confidence, Appearance, and the Total SEC index with MVC in Financial Well-Being, Friends, Family, and Love, which indicates the most significant modern adolescence values. The interrelationship of conflicts suggested that there were common predictors of Intra-Personal Conflicts, possibly accounting for personality and cognitive-evaluative PEW indices.

## Conclusion

This study highlighted the prevalence of Intra-Personal Conflicts in an adolescent sample. The respondents with high levels of intra-personal conflicts showed changes in all PEW indicators: personality, emotional, and cognitive-evaluative. Decreasing of personal indicators of PEW such as Environmental Mastery and Self-Acceptance is in correspondence with high level of MVC. A high level of SEC is characterized by decreasing of the most of PEW indicators: personal — decreasing of Cheerfulness and Active Attitude to Life Situation; cognitive-evaluative — decreasing of Life Satisfaction; emotional - decreasing of Stability, Emotional Tone, increasing of Despondency, Tension, Anxiety. The study showed that personality and cognitive-evaluative components of PEW played the part of conflict moderators, while emotional ones were manifested as effects of Intra-Personal Conflicts. A decline in Environmental Mastery and Self-Acceptance was accompanied by a high level of MVC. A high level of SEC was characterized by a decrease in the majority of PEW indicators. Adolescents with MVC were motivated by a desire for Personal Growth, whereas those manifesting SEC showed an absence of conflicts in Family, Friends, Freedom, Creativity, and Self-Confidence, which created the basis for the reliance on these spheres of life.

## Limitations

The study’s major limitation was the imbalance of the sample by gender with a predominance of girls and by having groups with a high level of Intra-Personal Conflicts. This can be partly explained by the literature on the greater severity of conflicts in adolescent girls (Fantalova, 2015; Golovey & Gruzdeva, 2017; Golovey at al., 2020). However, further gender-sensitive research is required.
